# Physical activity and internalization problems in middle school students: the chain mediating role of rumination thinking and peer acceptance

**DOI:** 10.1038/s41598-025-09202-9

**Published:** 2025-07-04

**Authors:** Kelei Guo, Shengyong Chen, Zhen Hui, Feng Guo, Jun Xiang, Dong Li

**Affiliations:** 1 School of Physical Education and Health, Zhaoqing University, Zhaoqing, China; 2 School of Marxism, Zhaoqing University, Zhaoqing, China; 3 School of Physical Education, Guang Dong Technology College, Zhaoqing, China

**Keywords:** Physical exercise, Middle school students, Internalizing problems, Rumination, Peer acceptance, Psychology, Human behaviour

## Abstract

This study explores methods to improve internalizing problems in middle school students through physical exercise, aiming to construct a mediational model that includes physical exercise, Rumination, peer acceptance, and internalizing problems. The model examines the interactions and influence pathways among these variables to provide a more comprehensive understanding of the mechanism through which physical exercise impacts internalizing problems in middle school students. 671 junior high school students were surveyed by physical exercise scale, Rumination scale, peer acceptance scale and internalization question scale. SPSS26.0 was used to analyze the data, followed by descriptive analysis, reliability analysis, Pearson correlation analysis, Harman single factor test and Bootstrap analysis. (1) Physical exercise was negatively correlated with internalizing problems (*r* = − 0.286), but the direct path between physical exercise and internalizing problems was not statistically significant (β = − 0.060, *p* > 0.05, CI [− 0.137, 0.018]); (2) Physical exercise was significantly negatively correlated with Rumination (β = − 0.174, *p* < 0.001, CI [− 0.255, − 0.094]) and significantly positively correlated with peer acceptance (β = 0.357, *p* < 0.001, CI [0.286, 0.429]). Rumination was significantly negatively correlated with peer acceptance (β = − 0.128, *p* < 0.001, CI [− 0.199, − 0.056]) and significantly positively correlated with internalizing problems (β = 0.289, *p* < 0.001, CI [0.222, 0.356]); peer acceptance was significantly negatively correlated with internalizing problems (β = − 0.372, *p* < 0.001, CI [− 0.448, − 0.296]). (3) The total effect of the chain mediation model was − 0.251, and the direct effect of physical exercise and internalized problems was − 0.059,The total effect accounted for 23.51%, Rumination and Peer acceptance played a mediating role between physical exercise and internalized behavior problems, and the total indirect effect size was − 0.192, The total effect accounted for 76.49%. The mediating effect values of Rumination and Peer acceptance between physical exercise and internalized behavior problems were − 0.050 and − 0.133, accounting for 19.92% and 52.99%, respectively. The chain-mediated effect between Rumination and Peer acceptance between physical exercise and internalized behavior problems was − 0.008, and the chain-mediated effect accounted for 3.19%. Physical exercise is negatively correlated with internalization, Rumination is negatively correlated with peer acceptance; Rumination and peer acceptance, as mediating variables, not only play an independent mediating role between physical exercise and junior high school students’ internalization problems, but also further strengthen their role in junior high school students’ internalization problems through chain mediation. Although the effect value of chain mediation is small, it is worthy of attention, suggesting that there is a synergistic effect among the variables. The results of this study provide an important reference for the design of school physical exercise curriculum. Through reasonable design of physical education curriculum, the interest and attraction of physical exercise can be increased, and the participation of students can be improved indirectly, thus promoting the improvement of internalization problems. At the same time, it can be considered to combine physical exercise with mental health education, and cultivate students’ methods and abilities to cope with pressure and negative emotions through physical exercise courses, cultivate positive and optimistic attitude, and improve junior middle school students’ mental toughness and social adaptability.

**Supplementary Information:**

The online version contains supplementary material available at 10.1038/s41598-025-09202-9.

## Introduction

The adolescent period is a crucial stage for the growth and development of life, especially in the middle school stage of junior high school students, the rapid development of physical and mental growth at the same time, with the physiological and psychological changes in adolescence, their psychological activities also gradually transition from relying on the family to independent exploration, and began to form their own life values and world views. From early adolescence, changes in the brain’s neuroendocrine processes lead to hormonal and morphological changes, which, while promoting physical, social, and psychological maturity, also greatly increase the risk of psychological health issues^1^. According to the World Health Organization’s 2019 report, about half of mental health problems emerge around the age of 14 during adolescence^2^. Data from China’s 2020 statistics show that approximately 40% of adolescents in middle school have mental health issues^3,4^. During life development, individuals may exhibit abnormal behaviors that hinder social adaptation, known as problem behaviors^5^. Achenbach was one of the first to classify problem behaviors into two categories: internalizing and externalizing problems^6^. Internalizing problems refer to symptoms that manifest internally, expressing pain within the individual, typically arising from excessive or unreasonable control of emotions, feelings, or cognition, such as depression and anxiety^7^.Studies have shown that the incidence of internalization problems among adolescents is 30-40%, while the incidence of externalization problems is about 15%^8^, which not only increases the risk of psychological disorders among junior high school students, but also affects the academic level and cognitive development of adolescents^5,9^.Therefore, whether from the perspective of preventing psychological disorders in middle school students or promoting their healthy development, exploring the occurrence, development, influencing factors, and adverse outcomes of internalizing problems in Chinese adolescents is of utmost importance.

A report from a UK health center indicates that school nurses play a crucial role in identifying mental health issues in children and adolescents. Their involvement can effectively prevent school teachers from misinterpreting symptoms of mental health problems as behavioral issues, thus preventing the worsening of mental health conditions and even the occurrence of severe events such as suicide^10^. In China, however, the implementation of a school mental health service system remains incomplete. Due to factors such as stigma and limited accessibility to psychological services, adolescents with mental health issues rarely seek medical help voluntarily^11^. Many middle school students, burdened with heavy academic pressures, also suffer from mental health problems^12^. Based on this, the present study attempts to explore the relationship between physical exercise and internalizing problems in middle school students from a psychological intervention perspective, examining the role of Rumination and peer acceptance in this mechanism, with the aim of providing insights for mental health interventions for middle school students.

### Physical exercise and internalizing problems

Physical exercise involves activities aimed at enhancing fitness, promoting physical and mental health, and improving or maintaining bodily functions. These activities may include fitness, recreation, health recovery, and mental-intellectual training^13^. Recent research has shown that physical exercise contributes to improving individuals’ physical, mental, and social adaptability, making it an effective method for maintaining overall well-being^14^. Internalizing problems refer to psychological issues that arise from excessive or unreasonable control over emotions, feelings, or cognition. Unlike externalizing problems, internalizing issues are often less visible to others and do not immediately harm or threaten others. However, they represent a long-term risk factor for mental health, commonly characterized by anxiety, depression, and loneliness^15^. Research has found that there is a significant negative correlation between physical exercise and internalizing problems such as depression and anxiety, and different forms of exercise may produce different effects^16,17^. Students who engage in moderate or higher levels of exercise report fewer internalizing problem behaviors than those with minimal exercise^18^. Therefore, we propose Hypothesis 1: Physical exercise is negatively correlated with internalizing problems.

### The mediating role of rumination

Rumination is a maladaptive response style, characterized by the repetitive thinking about negative events, their possible causes, and potential negative outcomes, without actively considering solutions to the problems themselves^19^. According to the response style theory, Rumination is a relatively stable and enduring personality trait that can trigger and exacerbate depressive emotions^20^. This mode of thinking is characterized by unconstructive circular thinking. When individuals face life adversity or emotional distress, they will fall into repeated self-reflection cycles, accompanied by the continuous accumulation of anxiety and depression^21^. Individuals affected by Rumination often lose their initiative to solve problems due to excessive focus on negative information, forming negative expectations for future social interactions, and thus leading to internalized psychological problems such as social avoidance behavior and loneliness^22^.Empirical studies have confirmed that Rumination is an important cognitive risk factor for depressive symptoms in adolescents and has a significant predictive effect on depression level^23^. Ruminative thinking is also a key influencing factor of anxiety. By strengthening cognitive processing of negative events, individuals’ anxiety intensity can be significantly enhanced^24^. In summary, the degree of Rumination was significantly positively correlated with the severity of internalization problems such as depression, loneliness and anxiety.

In research exploring the relationship between physical exercise and Rumination, a negative correlation has been found, suggesting that adolescents who engage in physical exercise exhibit lower levels of Rumination, Lack of physical exercise may result in higher levels of Rumination^25^. physical exercise has been shown to significantly reduce Rumination, Higher levels of physical exercise help reduce Rumination in adolescents, thereby lowering the risk of internalizing problems such as depression and anxiety^26^. Based on this evidence, we propose Hypothesis 2: Physical exercise can influence internalizing problems in middle school students through its effect on Rumination, and Rumination mediates the relationship between physical exercise and internalizing problems.

### The mediating role of peer acceptance

Peer acceptance refers to the extent to which an individual is accepted and valued within a peer group of similar age, reflecting the group’s attitude toward the individual and indicating the individual’s social status within the group. It also serves as a manifestation of an individual’s social abilities^27^. Studies show that adolescents spend approximately one-third of their time with peers^28^social support network shifts from the family system to the peer system, with peer support becoming the most important source of social support^29^.Peer acceptance, as a positive aspect of peer relationships, satisfies adolescents’ need for social interaction, effectively reduces stress, and alleviates negative emotions such as depression and anxiety that may arise from academic and social challenges^30^. It plays a buffering and protective role in the physical and mental health development of adolescents. In contrast, negative peer relationships can affect an individual’s social adaptability^31^academic performance^32^and increase the risk of internalizing problems such as depression^33^anxiety^33^and other emotional issues^34^.

The social function of physical exercise is closely related to the effect of psychological intervention. With the help of physical exercise, junior high school students can promote the emotional communication with their peer groups, strengthen the communication and interaction between them, and then comprehensively improve the level of individual physical and mental health, and effectively inhibit the generation of negative emotions. Studies have shown that physical exercise has a positive role in promoting individual peer relationship, and regular physical exercise has a significant effect on improving peer relationship^35^. Physical exercise was significantly positively correlated with peer acceptance in peer relationships, with students who exercised regularly scoring higher on peer acceptance than their peers who exercised occasionally^36^.The higher the level of peer acceptance, the lower the risk of internalizing problems. Therefore, physical exercise not only directly impacts internalizing problems but also indirectly affects them through peer acceptance. Based on this evidence, we propose Hypothesis 3: Peer acceptance mediates the relationship between physical exercise and internalizing problems in middle school students.

### The chain mediating role of rumination and peer acceptance

Rumination is a maladaptive thinking style in which individuals focus on themselves as a cognitive object, analyzing and evaluating the discrepancies between reality and ideal situations, leading to the escalation of negative emotions^37,38^. Previous research indicates a significant negative correlation between Rumination and peer relationships^39^. Adolescents, being in the puberty phase, experience emotional instability, and when they have negative emotional reactions, Rumination can amplify these responses, subsequently affecting their relationships with peers^40^. Peer acceptance, as a dimension of peer relationships, is considered a positive aspect of peer interactions. Recent studies suggest a close link between Rumination and peer acceptance. Higher levels of Rumination are associated with lower levels of peer acceptance^41^.

In research on chain mediation effects, studies have found that Rumination and self-compassion mediate the relationship between peer relationships and adolescent depression^42^. Similarly, peer acceptance and Rumination play a chain mediating role in the relationship between parenting styles and adolescents’ social anxiety^43^. While these studies did not directly address physical exercise, their findings support the idea that Rumination and peer acceptance mediate the relationship between psychological factors and internalizing problems in adolescents. These studies provide insight into the mechanisms of internalizing problems and potential interventions, considering multiple internal and external factors. Based on the rich body of previous research, this study proposes Hypothesis 4: Rumination and peer acceptance play a chain mediating role in the relationship between physical exercise and internalizing problems in middle school students.

**Fig. 1:**
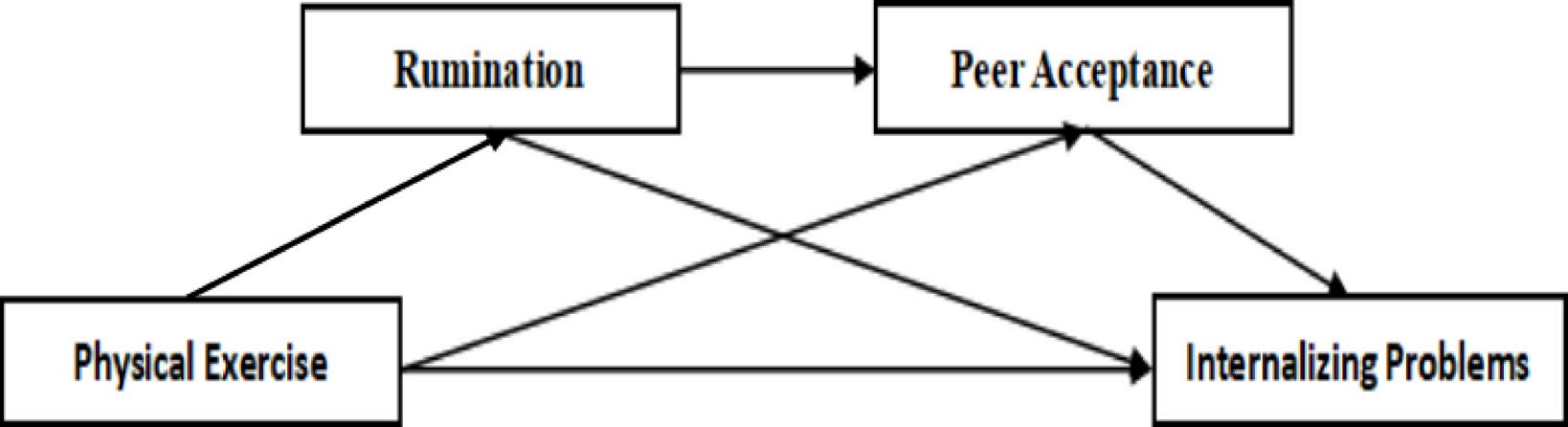
Conceptual framework diagram.

In summary, this study constructs a chained mediation model of the relationship between physical exercise and internalizing problems. From the perspective of physical exercise, it verifies the relationship mechanism between physical exercise and internalizing problems in middle school students and explores the chained mediating roles of Rumination and peer acceptance in this model (as shown in Fig. 1).

## Subjects and methods

### Subjects

A stratified cluster random sampling method was used to select one junior high school from both urban and rural areas in the southern, central, and northern regions of Guangning County for the survey. Stratified sampling was then conducted, randomly selecting two classes from each grade in both urban and rural schools, resulting in a total of 36 classes surveyed. A stratified cluster random sampling method was used to conduct a survey among middle school students in Zhaoqing City, Guangdong Province. A total of 800 survey questionnaires were distributed, and after excluding invalid and missing data, 671 valid questionnaires were collected, yielding an effective response rate of 83.88%. The participants’ ages ranged from 12 to 15 years, with a mean age of 13.99 ± 0.76 years. The sample consisted of 341 male and 330 female students, with 195 students in Grade 7, 344 students in Grade 8, and 132 students in Grade 9.

This study was conducted in accordance with the Declaration of Helsinki. All participants were informed about the purpose and nature of the study and provided written informed consent. Participation was voluntary, and the confidentiality of students’ information was ensured. Consent was obtained from both teachers and students during the testing process. For participants under 16 years of age, informed consent was additionally obtained from their parents or legal guardians.

### Measurements

#### Physical exercise

Physical exercise was measured using the college students’ physical exercise scale developed by Chen Shanping^44^ and revised by Wu Zhuyang^45^. The scale includes two dimensions: commitment to physical exercise and adherence to physical exercise, with 4 items in each dimension, totaling 8 items (e.g., “I often engage in physical exercise”). Responses were rated on a 5-point Likert scale (1 = strongly disagree, 5 = strongly agree), with the total score representing the level of physical exercise. Higher scores indicate a higher level of physical exercise. This scale has been used with Chinese middle school students^46^ and demonstrates high reliability and validity. In this study, the Cronbach’s α coefficient for this scale was 0.92.

#### Rumination

Rumination levels were assessed using the Rumination Questionnaire translated and revised by Chen Gongxing^47^. The scale includes two dimensions: reflective thinking (e.g., “Why do I have this problem when others do not?“) and compulsive thinking (e.g., “What should I do to cope with this emotion?“), with a total of 10 items. Responses were rated on a 4-point Likert scale (1 = almost never, 4 = almost always), and the average score of all items represents the total Rumination score. Higher scores indicate a higher level of Rumination. This scale has been used with Chinese middle school students^48^ and demonstrates high reliability and validity. In this study, the Cronbach’s α coefficient for this scale was 0.89.

#### Peer acceptance

Peer acceptance was measured using a Peer Acceptance Scale^49^which contains two items: “I get al.ong well with my peers” and “Peers seem to like me.” The scale uses a 7-point Likert scale (1 = strongly disagree, 7 = strongly agree), with the average score of the two items indicating the level of peer acceptance. Higher scores represent higher peer acceptance. This scale has been used with Chinese middle school students^50^ [46]. In this study, the Cronbach’s α coefficient for this scale was 0.82.

#### Youth self-report

The Youth Self-Report (YSR), developed by Achenbach^51^was used to assess internalizing symptoms in adolescents, specifically focusing on the anxiety/depression and withdrawal subscales. The scale consists of two dimensions: anxiety/depression (e.g., “I am overly fearful or worried”) and withdrawal (e.g., “I try to avoid close relationships with others”), with a total of 22 items. Responses were rated on a 3-point Likert scale (0 = not true, 2 = often true), with higher total scores indicating more severe internalizing symptoms. This scale has been used with Chinese middle school students^52^. In this study, the Cronbach’s α coefficient for this scale was 0.95.

### Statistical analysis

Data were analyzed using SPSS 26.0, performing common method bias testing, descriptive statistics, and correlation analysis. The PROCESS macro was used to test for chain mediation effects, with age and gender variables controlled for in the analysis.

## Results and analysis

### Common method bias test

A Harman single-factor test was used to check for common method bias in this study. The results showed that there were six factors with eigenvalues greater than 1, and the first factor explained 32.63% of the variance, which is less than the critical value of 40%. Therefore, it is concluded that there is no significant common method bias in this study.

### Descriptive statistics and correlation analysis

Independent sample t test was used to analyze the differences in physical exercise, Rumination, peer acceptance and internalization of junior middle school students of different genders. The results show that there are significant differences in the internalization problems of junior middle school students of different genders. There are differences in physical exercise, but the differences are not too significant; There were no differences in Rumination and peer acceptance (see Table 1).

**Table 1:** Gender differences among different variables, *N* = 671.

	Sex	Number	M ± SD	F	*P*
Physical exercise	Male	341	28.61 ± 6.20	4.02	0.045*
	Women	330	25.71 ± 5.90		
Rumination	Male	341	1.88 ± 0.62	0.82	0.365
	Women	330	2.02 ± 0.60		
Peer acceptance	Male	341	5.40 ± 1.54	3.21	0.073
	Women	330	4.94 ± 1.72		
Internalizing problems	Male	341	27.94 ± 7.36	39.78	0.000***
	Women	330	31.66 ± 9.45		

*p < 0.05, ***p < 0.001. (The same below).

The results of the correlation analysis (see Table 2) show that physical exercise is significantly correlated with Rumination, peer acceptance, and internalizing problems in all pairwise comparisons. Notably, the correlations between physical exercise, Rumination, and peer acceptance suggest that actively participating in physical exercise, reducing Rumination, and enhancing peer acceptance may help improve internalizing problems in middle school students. These findings provide preliminary support for our hypothesis.

**Table 2:** Correlation analysis of variables.

Variable	M ± SD	1	2	3	4	5	6
1.Gender	–	–					
2.Age	13.99 ± 0.76	− 0.74	1				
3.Physical exercise	27.18 ± 6.22	− 0.233**	0.051	1			
4.Rumination	1.95 ± 0.61	− 0.110**	0.022	− 0.189**	1		
5.Peer acceptance	5.17 ± 1.65	− 0.142**	− 0.011	0.391**	− 0.201**	1	
6.Internalizing problems	29.77 ± 8.65	− 0.215**	0.034	− 0.286**	0.389**	− 0.471**	1

* *p* < 0.05, ** *p* < 0.01, *** *p* < 0.001.

### Mediation analysis

According to Hayes^53^the nonparametric percentile bootstrap method was used to test the mediation effects using PROCESS (Version 3.3) Macro Model 6, with 5000 bootstrap samples and a 95% confidence interval (CI). The test results are shown in Table 3.

**Table 3:** Regression analysis between variables.

Result variable	Predictor	Fitting index			Coeffcient signifcance			Confidence Interval
		R	R^2^	F	β	t	*p*	
Rumination	Gender	0.204	0.042	8.83	0.145	1.876	0.061	− 0.007, 0 0.296
	Age				0.048	0.941	0.347	− 0.052, 0 0.147
	Physical exercis				− 0.174	− 4.262	0.000***	− 0.255, − 0.094
Peer Acceptance	Gender	0.415	0.172	40.24	− 0.092	− 1.229	0.219	− 0.240, 0 0.055
	Age				− 0.039	− 0.869	0.385	− 0.126, 0 0.049
	Physical exercis				0.357	9.839	0.000***	0.286, 0 0.429
	Rumination				− 0.128	− 3.517	0.000***	− 0.199, − 0.056
Internalizing Problems	Gender	0.576	0.331	46.74	0.238	3.646	0.000***	0.110, 0 0.366
	Age				0.046	1.134	0.257	− 0.034, 0.126
	Physical exercis				− 0.060	− 1.511	0.131	− 0.137, 0 0.018
	Rumination				0.289	8.492	0.000***	0.222, 0 0.356
	Peer acceptance				− 0.372	− 9.629	0.000***	− 0.448, − 0.296

* *p* < 0.05, ** *p* < 0.01, *** *p* < 0.001.

**Fig. 2:**
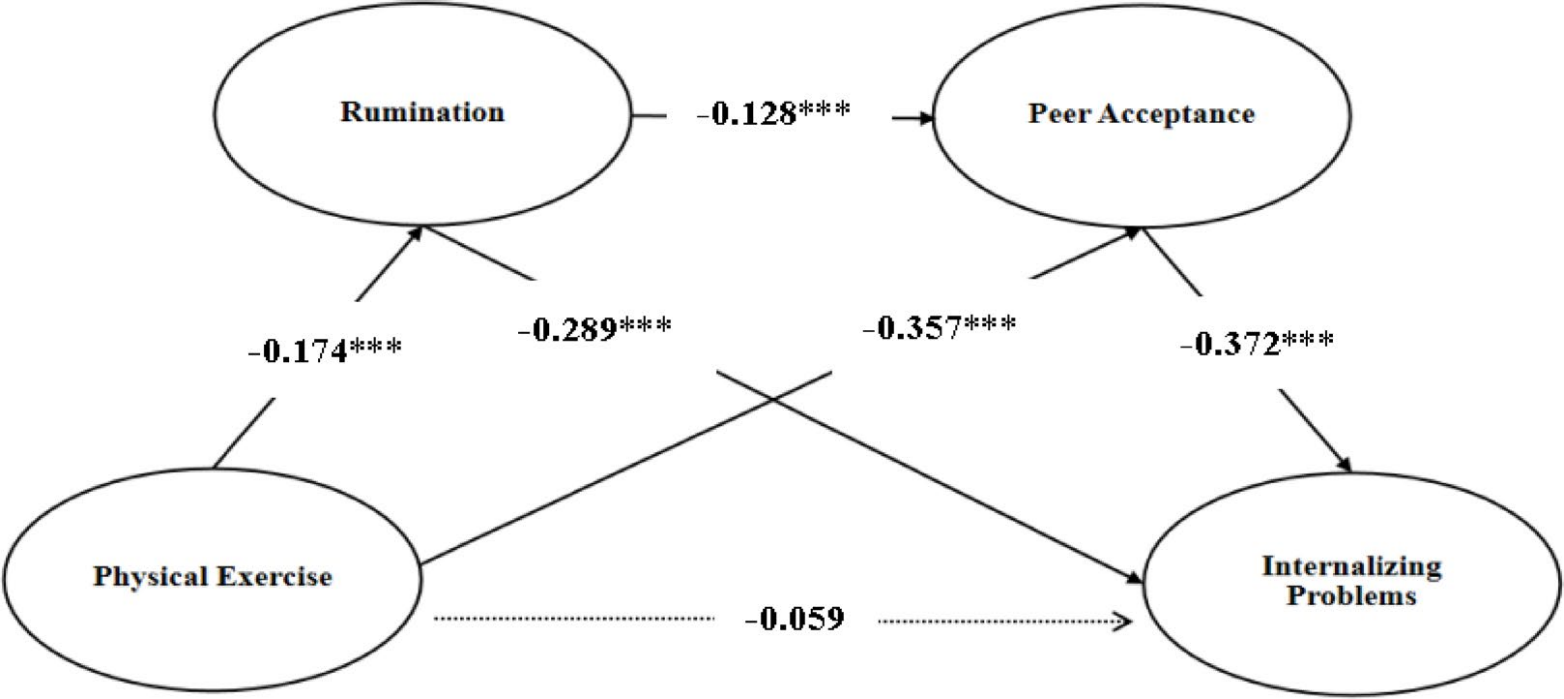
Mediation effect model.

First, we examined the direct path between physical exercise and internalizing problems in middle school students. The results showed a negative correlation between physical exercise and internalizing problems, Hypothesis 1 was supported.but the direct path was not significant (β = − 0.060, *p* > 0.05, CI [− 0.137, 0.018]), This may be due to the particularity of junior high school students as the object of this study and the limitations of the sample size, as well as the possible existence of unobserved mediating paths in the model. However, the chain mediating path fully explains the influence of physical exercise on internalization ‌, which is in line with the multi-level role theory of social psychological problems and reveals that we, The benefits of physical exercise should be realized through the synergistic transmission of psychological mechanisms, rather than acting independently. Next, we tested the mediating role of Rumination and peer acceptance in the relationship between physical exercise and internalizing problems (see Fig. 2). The results showed that physical exercise was significantly negatively correlated with Rumination (β = − 0.174, *p* < 0.001, CI [− 0.255, − 0.094]), and Rumination was significantly positively correlated with internalizing problems (β = 0.289, *p* < 0.01, CI [0.222, 0.356]). Therefore, Hypothesis 2 was supported.

Furthermore, physical exercise was significantly positively correlated with peer acceptance (β = 0.357, *p* < 0.001, CI [0.286, 0.429]), and peer acceptance was significantly negatively correlated with internalizing problems (β = − 0.372, *p* < 0.001, CI [− 0.448, − 0.396]). Thus, Hypothesis 3 was supported. Finally, Rumination was significantly negatively correlated with peer acceptance (β = − 0.128, *p* < 0.001, CI [− 0.199, − 0.056]), which supported Hypothesis 4. These findings suggest that Rumination and peer acceptance mediate the effect of physical exercise on internalizing problems, confirming the proposed mediation paths.

The mediation effect analysis results (see Table 2) show that the direct mediation effects of Rumination and peer acceptance are significant. Furthermore, the chain mediation effect involving both Rumination and peer acceptance is also significant.

The bias-corrected percentile Bootstrap method (with 5,000 resamples) was used for testing. As shown in Table 4, The total effect size is − 0.251, 95% confidence interval (CI) is [− 0.333, − 0.169], and the direct effect is − 0.059, confidence interval is [− 0.137,0.018], The total effect accounted for 23.51%, The total indirect effect is − 0.192 with confidence intervals [− 0.245, − 0.147], The total effect accounted for 76.49%.Among them, the CI for physical exercise → Rumination→ internalizing problems was [− 0.077, − 0.025], with a mediation effect size of − 0.050, The total effect accounted for 19.92%; the CI for physical exercise → peer acceptance → internalizing problems was [− 0.172, − 0.101], with a mediation effect size of − 0.133, The total effect accounted for 52.99%; and the CI for physical exercise → Rumination→ peer acceptance → internalizing problems was [− 0.017, − 0.003], with a mediation effect size of − 0.008, The total effect accounted for 3.19%. Since none of the confidence intervals included zero, the mediation effects were statistically significant. The mediation effects of Rumination and peer acceptance between physical exercise and subjective well-being are illustrated in Fig. 2.

**Table 4:** Proportion of the mediating effect.

Influence path	Effect size	SE	Proportion	Confidence interval	
				LLCI	ULCI
Total effect	− 0.251	0.041	100%	− 0.333	− 0.169
Direct effect	− 0.059	0.039	23.51%	− 0.137	0.018
Physical exercise → rumination→ internalizing problems	− 0.050	0.013	19.92%	− 0.077	− 0.025
Physical exercise → peer acceptance → internalizing problems	− 0.133	0.018	52.99%	− 0.172	− 0.101
Physical exercise → rumination→ peer acceptance → internalizing problems	− 0.008	0.003	3.19%	− 0.017	− 0.003
Total indirect effect	− 0.192	0.024	76.49%	− 0.245	− 0.147

## Discussion

### Physical exercise and internalizing problems

The results of this study indicate a significant negative correlation between physical exercise and internalizing problems, consistent with previous findings, supporting Hypothesis 1.However, due to the particularity of the psychological and behavioral development of junior high school students, the regression analysis results show that the predictive effect of physical exercise on the internalization problems of junior high school students has not reached a significant level. The psychological development of junior high school students is not mature, and their emotions fluctuate greatly, which is easy to be affected by many factors. For example, family environment, school atmosphere, academic pressure, etc. may have an important impact on internalization problems, and under the influence of these factors, the direct effect of physical exercise on internalization problems may be relatively weak. The emergence of internalization problem is a complex psychological process, which is influenced by many factors. Physical exercise may be only one of many influencing factors, and its effect may be regulated and mediated by other psychological factors. For example, individual cognitive style, coping style and personality traits may affect the effect of physical exercise on internalization problems. If these factors vary widely in the sample, the direct effect of physical exercise on internalization problems may be masked.

### Mediating role of rumination

The results of this study show that physical exercise is significantly negatively correlated with Rumination, and Rumination is significantly positively correlated with internalizing problems. This aligns with previous findings, confirming that Rumination mediates the relationship between physical exercise and internalizing problems. The mediating effect is significant, thus supporting Hypothesis 2.

Firstly, physical exercise is widely recognized as having a positive impact on mental health. It helps regulate negative emotions, improves physical and mental states, alleviates psychological stress, and plays a crucial role in maintaining a healthy mindset. While research directly linking physical exercise to Rumination is relatively scarce, this study provides new evidence for such a relationship. The findings indicate that higher levels of physical exercise are associated with lower levels of Rumination among middle school students, consistent with existing research^54^. Adolescents who lack physical exercise tend to exhibit higher levels of Rumination. Physical exercise serves as a positive coping strategy, helping middle school students shift their focus, enhance self-efficacy, and improve their psychological well-being by reducing Rumination.

Secondly, Rumination has been shown to positively predict internalizing problems, such as depression, loneliness, and anxiety. This result aligns with the core tenets of the response styles theory, which posits that individuals with ruminative thinking styles tend to fixate on their negative emotions and circumstances when facing adverse events. This habitual overthinking exacerbates negative self-perception, amplifies the detrimental impact of negative emotions, and increases the risk of depression and anxiety^55^. Middle school students with high levels of Rumination are more likely to exhibit negative emotions towards people and situations. They adopt maladaptive thought patterns and are less inclined to engage in positive interactions with peers and teachers, resulting in heightened feelings of loneliness and social anxiety^56^. Consequently, adolescents with higher levels of Rumination display more internalizing problems.

Finally, this study examined the mediating role of Rumination between physical exercise and internalization problems, and the results showed that after adding Rumination as a variable, physical exercise could still predict various internalization problems, and could also predict internalization problems through Rumination, and Rumination thought played a partial mediating role.The mediating effect is significant, accounting for 19.92%, suggesting that physical exercise, as a positive coping strategy, can help junior middle school students to free themselves from Rumination and reduce the occurrence of Rumination by promoting physical and mental health, releasing pressure and improving mood, thereby reducing the repetition and repeated thinking caused by adverse emotions. Reduce anxiety, depression and other internalized problems of junior high school students.

### Mediating role of peer acceptance

The results of this study show that physical exercise is significantly positively correlated with peer acceptance, and peer acceptance is significantly negatively correlated with internalizing problems. These findings are consistent with previous research, confirming that peer acceptance mediates the relationship between physical exercise and internalizing problems. The mediating effect is significant, thus supporting Hypothesis 3.

Adolescents who frequently participate in physical exercise are often immersed in a positive exercise environment, which subtly influences their behavior. Over time, their interactions and cooperation with peers increase, fostering the development of good peer relationships^57^. There is a mutually reinforcing relationship between physical exercise and peer acceptance. As a social interaction activity, physical exercise allows middle school students to showcase their abilities, enhancing their image and status among peers. This process cultivates positive qualities such as confidence, resilience, and optimism, which often make them more likable among their peers, leading to greater peer acceptance. In turn, peer acceptance further motivates students to participate in physical exercise actively, creating a positive feedback loop that is crucial for adolescents’ holistic development.

On the other hand, peer acceptance negatively predicts internalizing problems. According to the “Person-Context Interaction Theory,” psychological development is influenced by both environmental and individual factors^58^. For middle school students, in addition to environmental influences, interpersonal interactions play a crucial role in mental health. Peer acceptance is not only a key predictor of mental health but also influences how individuals adapt interpersonally and regulate emotions when facing stress^59^. Adolescents with high levels of peer acceptance demonstrate better emotional regulation and are more likely to receive emotional support during stressful events^60^. In contrast, low levels of peer acceptance impair social functioning, making it difficult for adolescents to manage negative emotions and stress, thereby increasing the risk of internalizing problems such as depression and anxiety^61^.

Finally, as a key variable in this study, peer acceptance was tested for its mediating role in the relationship between physical exercise and internalizing problems. The results indicate that physical exercise not only directly affects internalizing problems but also influences them indirectly through peer acceptance. It is worth noting that the mediating effect of peer acceptance accounts for 68.42%, indicating its central role in the mental health of junior high school students. Physical exercise should not be regarded as an isolated psychological intervention, but should become an important fulcrum to build a positive social support network.Physical exercise enhances individual interaction and cooperation with peers, increasing levels of peer acceptance and fostering the formation of positive peer relationships. These relationships provide emotional support and social recognition, alleviating stress, anxiety, and depression, thereby reducing internalizing problems in middle school students.

### Chain mediation role of rumination and peer acceptance

The results of this study show that Rumination is significantly negatively correlated with peer acceptance, consistent with previous findings. To further analyze the relational mechanism between Rumination, peer acceptance, physical exercise, and internalizing problems, a chain mediation model was constructed. The results indicate that Rumination and peer acceptance jointly mediate the relationship between physical exercise and internalizing problems, forming a chain mediation effect. Hypothesis 4 is thus supported.

Response styles theory suggests that Rumination not only exacerbates depressive emotions but also disrupts problem-solving by reinforcing negative thought patterns. As such, Rumination is both a risk factor for internalizing problems such as depression and anxiety and a critical target for interventions aimed at mitigating these issues. Reducing Rumination and fostering mindfulness can help alleviate symptoms of depression and anxiety^62^.Ruminative thinking is easy to cause junior high school students to show negative and sensitive emotions and behaviors in peer communication. They may focus too much on their own negative emotions and experiences and avoid deep communication and interaction with their peers, thus reducing the chances of acceptance by their peers. This kind of social avoidance behavior not only makes middle school students miss valuable opportunities to establish and maintain peer relationships, but also may aggravate their loneliness and social anxiety. Rumination may also cause junior high school students to transmit negative emotions in peer communication. Their negative emotions and behaviors may infect other peers, making the atmosphere of the entire peer group tense and depressing. This kind of emotional contagion will not only affect the harmony and stability of the peer relationship, but also aggravate the internalization problem of junior high school students.

Adolescence is a critical period of individual physical and mental development. Physically, it experiences puberty development, and the secretion of sex hormones leads to drastic emotional fluctuations. Psychologically, it faces the conflict between self-identity and chaotic role. At this stage, individuals’ demand for peer relationship is significantly enhanced, and peer acceptance becomes the core index to measure social adaptability. Good peer acceptance relationships can provide positive emotional support and a sense of belonging to junior high school students, help them better cope with challenges and pressures in life, and reduce the incidence of internalization problems. Conversely, inadequate peer acceptance may lead middle school students to feel lonely and helpless, increasing the risk of internalizing problems.By actively participating in physical exercise, middle school students develop a more optimistic and positive outlook, reduce unnecessary repetitive thinking, and divert their attention away from excessive self-focus. This decrease in Rumination fosters better interpersonal skills, enhancing peer acceptance and reducing the likelihood of internalizing problems. Thus, physical exercise contributes to improving psychological well-being by breaking the chain of negative cognition and social withdrawal through its positive impact on both Rumination and peer relationships.

### Research significance

This study systematically analyzes and combs the empirical studies on junior high school students’ physical exercise, Rumination, peer acceptance and other aspects, elaborates the multiple factors affecting the internalization problem, provides a new perspective for intervening in the internalization problem of junior high school students, and expands the research path to solve the internalization problem of junior high school students. This paper has some practical effects on individual mental health and behavior adaptation to guide junior high school students to participate in sports actively, face learning pressure and life events correctly, and establish good peer relationship. At the same time, schools and physical education educators should pay attention to the role of physical exercise in promoting students’ mental health, and encourage students’ active participation by organizing diversified sports activities to improve their internalization problems. For middle school students with internalization problems, psychological intervention can be implemented by reducing Rumination and enhancing peer acceptance. For example, cognitive behavioral training helps students change negative thinking patterns, team building activities enhance students’ social ability and sense of peer acceptance, and establish good peer relationships to reduce the risk of depression, anxiety and other internalized problems of junior high school students.

### Limitations and future prospects

Although this study has achieved certain results, there are still some shortcomings in the whole process, which need to be improved in the future research. This study uses questionnaire survey for self-report, which may be favorable to some extent and affect the rigor of this study. In the future, data can be collected by combining other people’s evaluation and self-report. The sample was mainly derived from middle school students in a specific area, which may limit the generalizability of the results. Future studies should expand the sample scope to include students from different regions and different age groups to improve the representativeness and external validity of the study. This study focused on the relationship between physical exercise, Rumination, peer acceptance, and internalization problems, but there may be other variables that were not included that influenced the results. Future studies should further control for other potential variables to more accurately reveal the relationship between the variables. This study uses a cross-sectional design and cannot determine a causal relationship between variables. Future studies should adopt longitudinal design or experimental design to explore the dynamic relationship and causality between variables in more depth.

## Conclusion

This study found that physical exercise is negatively correlated with internalizing problems in middle school students, while Rumination is negatively correlated with peer acceptance. Rumination plays an independent mediating role in the relationship between physical exercise and internalizing problems, as does peer acceptance. Additionally, Rumination and peer acceptance together form a chain mediation effect, further elucidating the mechanisms by which physical exercise influences internalizing problems in adolescents.

## Electronic supplementary material

Below is the link to the electronic supplementary material.

Supplementary Material 1.

## Data Availability

All datasets generated for this study are included in the article/supplementary material.
