# Correction: Physical activity and internalization problems in middle school students: the chain mediating role of rumination thinking and peer acceptance

**DOI:** 10.1038/s41598-025-16372-z

**Published:** 2025-08-20

**Authors:** Kelei Guo, Shengyong Chen, Zhen Hui, Feng Guo, Jun Xiang, Dong Li

**Affiliations:** 1 School of Physical Education and Health, Zhaoqing University, Zhaoqing, China; 2 School of Marxism, Zhaoqing University, Zhaoqing, China; 3 School of Physical Education, Guang Dong Technology College, Zhaoqing, China

Correction to: *Scientific Reports* 10.1038/s41598-025-09202-9, published online 04 July 2025

The original version of this Article contained an error in the spelling of the author Kelei Guo which was incorrectly given as Keilei Guo.

The original Article has been corrected.

