# Supplementary material for: Physical activity and internalization problems in middle school students: the chain mediating role of rumination thinking and peer acceptance
Source: Sci Rep. 2025 Jul 4;15:23976. doi: 10.1038/s41598-025-09202-9 (PMC12227635; doi:10.1038/s41598-025-09202-9)
Supplement: Supplementary file 1 — Supplementary Material 1. [file 41598_2025_9202_MOESM1_ESM.docx]

**Physical Activity and Internalization Problems in Middle School Students: the Chain Mediating Role of Rumination Thinking and Peer Acceptance**

Contents

[Appendix A Volunteer informed consent 2](#_Toc18765)

[Appendix B Questionnaire on Physical Activity and Internalization for Middle School Students 4](#_Toc4877)

# **Appendix A Volunteer informed consent**

**Volunteer informed consent**

1. **Project name:** Physical Activity and Internalization Problems in Middle School Students: the Chain Mediating Role of Rumination Thinking and Peer Acceptance

**2. Research purpose:**

First, this study will explore ways to improve junior high school students' internalization problems from the perspective of physical exercise, construct a mediation model that includes physical exercise, rumination thinking, peer acceptance, and internalization problems, and comprehensively examine the interactions and paths of influence among the variables in order to more comprehensively understand the mechanism of physical exercise's influence on junior high school students' internalization problems. Second, this study used the Physical Activity Scale, the Rumination Thinking Scale, the Peer Acceptance Scale, and the Internalization Problem Scale to conduct a questionnaire survey with 671 junior high school students. SPSS 26.0 was used to analyze the data, and Pearson correlation analysis, structural equation modeling test, and bias-corrected percentile bootstrap were performed sequentially. We are glad that you can cooperate with us and thank you for your contribution to sports.

**3. Impact on you:**

Only questionnaire filling, only for scientific research, no negative impact on you, will not make the data public.

**4. Concept explanation:**

Physical exercise is a physical activity that uses physical practice and exercise load as a means of fitness and bodybuilding, recreation and leisure, health care and rehabilitation, and mental intelligence exercise as the content of the activity, in order to enhance physical fitness, improve physical and mental health, and improve and maintain the ability of the organism.

Internalization problems are a class of symptoms that point to the interior of an individual and express pain inwardly, symptoms that arise from excessive or unreasonable control of one's emotions, moods, or perceptions, such as depression, anxiety, and so on.

Ruminate thinking is a form of maladaptive response that can be described as when a person, after experiencing some negative event, repeats thoughts about the event as well as possible causes and potential negative outcomes, but does not actively think about how to solve the problem itself.

Peer acceptance refers to the degree to which an individual is accepted and noticed in age-matched or peer group relationships, which can reveal a group's attitude toward an individual, and also reflects an individual's status among peers, which is a manifestation of an individual's social competence.

**5. Commitment:**

The above research data is only for scientific research writing, and the original data will not be disclosed and publicized.

In addition, when filling in the data, we will replace your real name with English letters to achieve confidentiality.

If you have any questions about the above terms, you can directly consult our staff. By signing below, you indicate that you have read and understood the above terms, and agree to participate in the above study.

Signature of the volunteer (or legal guardian) :

This project research group

Year month day

# Appendix B Questionnaire on Physical Activity and Internalization for Middle School Students

**Questionnaire on Physical Activity and Internalization for Middle School Students**

**Dear Students:**

Greetings! This questionnaire may take about 3 minutes of your time! The purpose of this survey is to understand the relationship between physical activity and internalization problems among middle school students. The results of the survey will be used for scientific research only, and the answers will not be considered correct or incorrect, so you can feel free to choose the answers that match your actual situation. Thank you for your support and cooperation! Good luck in your studies and have a happy life!

**I. General Information Questionnaire**

1. your gender ① male ② female

2. your age ①12 ②13 ③14 ④15

3. Your grade level ①First year ②Second year ③Third year ④Have you ever been left behind?

4. Have you ever stayed behind ①have stayed behind = 1, ②no stayed behind experience

**Ⅱ. Physical activity scale (Likert 5 scale, strongly disagree", "disagree", "neither agree nor disagree", "agree" and "strongly agree") agree", "disagree", "neither agree nor disagree", "agree" and "strongly agree" on a scale of 1 to 5)**

1. It is difficult for me to withdraw from physical exercise.

2. there is a strong desire to participate in physical activity after a few days of not exercising

3. it is difficult for me to accept a lifestyle that lacks physical activity.

4. physical activity is an integral part of my life.

5. I am better at staying physically active.

6. I hardly ever stop taking physical exercise and I am able to stick to it for a long period of time. 7.

7. I have the habit of exercising. 8.

8. I regularly participate in physical exercise.

**III. Ruminate Thinking Scale (1-4 points, hardly ever, sometimes, often, almost always)**

**What do you usually do when you feel lost, sad or depressed?**

1. think "What can I do to cope with this emotion? "

2. analyze recent events to try to understand why you are depressed .

3. think "Why do I always react this way? "

4. go out alone and think about why you feel this way .

5. write down and analyze your thoughts.

6. think about how things have been going lately and hope that it will go in a good direction .

7. think "Why do I have this problem and others don't? "

8. think "Why can't I handle things better? "

9. Analyze your personality to try to understand why you are depressed.

10. Go to a place alone to think about your feelings.

**Peer Acceptance Scale (Likert 7-point scale, totally disagree Disagree Comparatively disagree Fairly agree Comparatively agree Agree Totally agree)**

1. I get along well with those my own age.

2. people my age seem to like me.

**V. Adolescent Self-Rating Scale (Likert 3-point scale, None Sometimes Often)**

1. I feel lonely.

2. I cry and scream a lot.

3. I hurt myself on purpose or attempt suicide.

4. I am afraid I will have bad thoughts or do bad things.

5. I feel I have to be perfect.

6. I feel that no one likes me.

7. I feel that people are trying to make fun of me.

8. I feel worthless or have an inferiority complex.

9. I prefer to be by myself rather than with others.

10. I am nervous, easily agitated or tense.

11. I am excessively fearful or worried.

12. I feel excessively guilty.

13. I refuse to talk to people.

14. I keep my mouth shut and don't talk about things.

15. I am easily embarrassed or feel unnatural.

16. I am shy.

17. I am suspicious.

18. I have thought about suicide.

19. I have low energy.

20. I am sullen, sad or depressed.

21. I try to avoid deep friendships.

22. I worry a lot.
